# Supplementary material for: Splice donor site sgRNAs enhance CRISPR/Cas9-mediated knockout efficiency
Source: PLoS One. 2019 May 9;14(5):e0216674. doi: 10.1371/journal.pone.0216674 (PMC6508695; doi:10.1371/journal.pone.0216674)
Supplement: S1 Table — NGS analysis of allelic variants induced in Baf/3 mouse cells. (DOCX) [file pone.0216674.s001.docx]

**S1 Table**.- *In vitro* genome editing of the mouse *Tyr* locus using sgRNA against exon coding sequence (IE) and the coding splice-donor exon (SDE) sequence. NGS analysis of allelic variants induced in Baf/3 mouse cells.

| **IE*-mTyr* sgRNA** | **Sequence** | **Mutation** | **Result** | **Protein translation** |
| --- | --- | --- | --- | --- |
| **WT** | TTTATAATAGGACCTGCCAGTGCTCAGGCAACTTCATGGGTTTCAACTGC |  |  |  |
| **Ins C** | TTTATAATAGGACCTGCCAGT**C**GCTCAGGCAACTTCATGGGTTTCAACTGC | Frameshift +1 bp | Stop | No |
| **Del G** | TTTATAATAGGACCTGCCAGT--CTCAGGCAACTTCATGGGTTTCAACTGC | Frameshift -1 bp | Stop | No |
| **Del C** | TTTATAATAGGACCTGCCAGTGC----AGGCAACTTCATGGGTTTCAACTGC | Frameshift -2 bp | Stop | No |
| **Del TC** | TTTATAATAGGACCTGCCAGTGC----AGGCAACTTCATGGGTTTCAACTGC | Frameshift -2 bp | Stop | No |
| **Del TGCTCA** | TTTATAATAGGACCTGCCAG-----------GGCAACTTCATGGGTTTCAACTGC | In frame -6 bp | CS/-- | Yes |
| **Del ACCTGCCAGTGCTCAGG** | TTTATAATAGG--------------------------------CAACTTCATGGGTTTCAACTGC | Frameshift -17 bp | Stop | No |
| **Del GCCAGTGCTCAGGCAACT** | TTTATAATAGGACCT-------------------------------CTTCATGGGTTTCAACTGC | In frame -18 bp | QCSGNF/-- | Yes |
| **Del TGCCAGT** | TTTATAATAGGACC-------------GCTCAGGCAACTTCATGGGTTTCAACTGC | Frameshift -7 bp | Stop | No |
| **Del AGTGC** | TTTATAATAGGACCTGCC----------TCAGGCAACTTCATGGGTTTCAACTGC | Frameshift -5 bp | Stop | No |
| **SDE*-mTyr sgRNA*** | **Sequence (Splice site; Exon; Intron)** | **Mutation** | **Result** | **Protein translation** |
| **WT** | AGCCCAGCATCCTTCTTCTCCTCCTGGCAGGTAAGATGCACTATATAGAG |  |  |  |
| **Ins A** | AGCCCAGCATCCTTCTTCTCCTCCTGGCA**A**GGTAAGATGCACTATATAGAG | Frameshift +1 bp | Stop | No |
| **Del G** | AGCCCAGCATCCTTCTTCTCCTCCTGGCAG--TAAGATGCACTATATAGAG | Sp donor site -1 bp |  | No |
| **Del TAA** | AGCCCAGCATCCTTCTTCTCCTCCTGGCAG--------GATGCACTATATAGAG | Sp donor site -3 bp |  | No |
| **Del TAAG** | AGCCCAGCATCCTTCTTCTCCTCCTGGCAGG--------ATGCACTATATAGAG | Sp donor site -4 bp |  | No |
| **Del GT** | AGCCCAGCATCCTTCTTCTCCTCCTGGCAG----AAGATGCACTATATAGAG | Sp donor site -2 bp |  | No |
| **Del GGTA** | AGCCCAGCATCCTTCTTCTCCTCCTGGCA--------AGATGCACTATATAGAG | Frameshift -1bp/ Sp donor site -4 bp | Stop | No |
| **Del AGG** | AGCCCAGCATCCTTCTTCTCCTCCTGGC-------TAAGATGCACTATATAGAG | Frameshift -2bp / Sp donor site -3 bp | Stop | No |
| **Del AG** | AGCCCAGCATCCTTCTTCTCCTCCTGGC-------TAAGATGCACTATATAGAG | Frameshift -2bp / Sp dpnor site -2 bp | Stop | No |
| **Del GTAAG** | AGCCCAGCATCCTTCTTCTCCTCCTGGCAG-----------ATGCACTATATAGAG | Sp donor site -4 bp |  | No |
