# Supplementary material for: Splice donor site sgRNAs enhance CRISPR/Cas9-mediated knockout efficiency
Source: PLoS One. 2019 May 9;14(5):e0216674. doi: 10.1371/journal.pone.0216674 (PMC6508695; doi:10.1371/journal.pone.0216674)
Supplement: S2 Table — NGS analysis of allelic variants induced in K562 human cells. (DOCX) [file pone.0216674.s002.docx]

**S2 Table**. - *In vitro* genomic edition of human *TYR* locus using sgRNA against exon coding sequence (IE) and coding SDE sequence. NGS analysis of allelic variants induced in K562 human cells.

| **IE-*hTYR* sgRNA** | **Sequence** | **Mutation** | **Result** | **Protein translation** |
| --- | --- | --- | --- | --- |
| **WT** | ATGCATTATTATGTGTCAATGGATGCACTGCTTGGGGGATCTGAAATCTG |  |  |  |
| **Ins t** | ATGCATTATTATGTGTCAATGGATGCAC**T**TGCTTGGGGGATCTGAAATCTG | Frameshift | Stop | No |
| **Del TGCAC** | ATGCATTATTATGTGTCAATGGA-----------TGCTTGGGGGATCTGAAATCTG | Frameshift | Stop | No |
| **A-T** | ATGCATTATTATGTGTCAATGGATGC**T**CTGCTTGGGGGATCTGAAATCTG | In frame |  | Yes |
| **Del CA** | ATGCATTATTATGTGTCAATGGATG----CTGCTTGGGGGATCTGAAATCTG | Frameshift | Stop | No |
| **Del C** | ATGCATTATTATGTGTCAATGGATGCA--TGCTTGGGGGATCTGAAATCTG | Frameshift | Stop | No |
| **SDE-*hTYR* sgRNA** | **Sequence (Splice site; Exon; Intron)** | **Mutation** | **Result** | **Protein translation** |
| **WT** | AGCCCAGCATCATTCTTCTCCTCTTGGCAGGTAAGATATGCTAGATATAC |  |  |  |
| **Ins T** | AGCCCAGCATCATTCTTCTCCTCTT**T**GGCAGGTAAGATATGCTAGATATAC | Frameshift +1 bp | Stop | No |
| **Del G** | AGCCCAGCATCATTCTTCTCCTCTT--GCAGGTAAGATATGCTAGATATAC | Frameshift -1 bp | Stop | No |
| **Del CTTG** | AGCCCAGCATCATTCTTCTCCT-------GCAGGTAAGATATGCTAGATATAC | Frameshift -4 bp | Stop | No |
| **Del TGGCAGG** | AGCCCAGCATCATTCTTCTCCTCT--------------TAAGATATGCTAGATATAC | In frame -6 bp / Sp donor site -4 bp | WQ/-- | No |
| **Del T** | AGCCCAGCATCATTCTTCTCCTC--TGGCAGGTAAGATATGCTAGATATAC | Frameshift -1 bp | Stop | No |
