# Supplementary material for: Splice donor site sgRNAs enhance CRISPR/Cas9-mediated knockout efficiency
Source: PLoS One. 2019 May 9;14(5):e0216674. doi: 10.1371/journal.pone.0216674 (PMC6508695; doi:10.1371/journal.pone.0216674)
Supplement: S3 Table — NGS analysis of allelic variants induced in K562 human cells. (DOCX) [file pone.0216674.s003.docx]

**S3 Table.-** *In vitro* genome editing of the human *ATM* locus using sgRNA against the exon coding sequence (IE) and the coding SDE sequence. NGS analysis of allelic variants induced in K562 human cells.

| **IE-*hATM*sgRNA** | **Sequence** | **Mutation** | **Result** | **Protein translation** |
| --- | --- | --- | --- | --- |
| **WT** | ACAGCGACATGGGGAACGTACACCATATGTGTTACGATGCCTTACGGAAG |  |  |  |
| **Ins G** | ACAGCGACATGGGGAACGTACACCATATGGTGTTACGATGCCTTACGGAAG | Frameshift +1 bp | Stop | No |
| **Del TG** | ACAGCGACATGGGGAACGTACACCATA -- TGTTACGATGCCTTACGGAAG | Frameshift -2 bp | Stop | No |
| **Del AT** | ACAGCGACATGGGGAACGTACACC -- ATGTGTTACGATGCCTTACGGAAG | Frameshift -2 bp | Stop | No |
| **G – A** | ACAGCGACATGGGGAACGTACACCATATATGTTACGATGCCTTACGGAAG | In frame | V/M | Yes |
| **Del TATG** | ACAGCGACATGGGGAACGTACACCA ------ TGTTACGATGCCTTACGGAAG | Frameshift -4 bp | Stop | No |
| **Del ACCATATG** | ACAGCGACATGGGGAACGTAC --------------TGTTACGATGCCTTACGGAAG | Frameshift -8 bp | Stop | No |
| **Del ATGTGTTACG** | ACAGCGACATGGGGAACGTACACCAT------------------ATGCCTTACGGAAG | Frameshift -10 bp | Stop | No |
| **Del GTACACCATAT** | ACAGCGACATGGGGAAC ------------------- GTGTTACGATGCCTTACGGAAG | Frameshift -11 bp | Stop | No |
| **T – G** | ACAGCGACATGGGGAACGTACACCATATGGGTTACGATGCCTTACGGAAG | In frame | V/G | Yes |
| **Del CCATAT** | ACAGCGACATGGGGAACGTACA-----------GTGTTACGATGCCTTACGGAAG | In frame -6 bp | PY/-- | Yes |
| **Del CATATGTGTTACGATGCCTTA** | ACAGCGACATGGGGAACGTACAC--------------------------------------CGGAAG | In frame -21 bp | YVLRCT/------ | Yes |
| **Del ACGTACACCATATGTGTTACGATGCCTT** | ACAGCGACATGGGGA---------------------------------------------------ACGGAAG | Frameshift -28 bp | Stop | No |
| **Del TGGGGAACGTACACCATATG** | ACAGCGACA--------------------------------------TGTTACGATGCCTTACGGAAG | Frameshift -20 bp | Stop | No |
| **SDE-*hATM*sgRNA** | **Sequence (Splice site; Exon; Intron)** | **Mutation** | **Result** | **Protein translation** |
| **WT** | ATTTACTGGGTCAGCCTGCAGACCTTCATGGTAAGTTCAGCATGCATTAT |  |  |  |
| **Ins T** | ATTTACTGGGTCAGCCTGCAGACCTTCA**T**TGGTAAGTTCAGCATGCATTAT | In frame / Sp donor site | C/L | No |
| **Del TTCATGGTAAG** | ATTTACTGGGTCAGCCTGCAGACC--------------------TTCAGCATGCATTAT | In frame -6 bp / Sp donor site -7 bp | SC/-- | No |
| **Ins A** | ATTTACTGGGTCAGCCTGCAGACCTTC**A**ATGGTAAGTTCAGCATGCATTAT | Frameshift +1 bp | Stop | No |
| **Del GTCAGCCTGCAGACCTTCATGGTAAGT** | ATTTACTGG-------------------------------------------------TCAGCATGCATTAT | In frame -21 bp / Sp donor Site -7 bp |  | No |
| **T-G** | ATTTACTGGGTCAGCCTGCAGACCTTCA**G**GGTAAGTTCAGCATGCATTAT | In frame / Sp donor site | W/G | No |
| **Del CA** | ATTTACTGGGTCAGCCTGCAGACCTT----TGGTAAGTTCAGCATGCATTAT | Frameshift -2 bp / Sp donor site -2 bp |  | No |
| **Del TCA** | ATTTACTGGGTCAGCCTGCAGACCT------TGGTAAGTTCAGCATGCATTAT | In frame -3 bp / Sp donor site -2 bp | S/- | No |
| **Del TGCAGACCTTCA** | ATTTACTGGGTCAGCC-----------------------TGGTAAGTTCAGCATGCATTAT | In frame -12 bp / Sp donor site -1 bp | CRPSC/----- | No |
| **Del ACCTTCA** | ATTTACTGGGTCAGCCTGCAG-------------TGGTAAGTTCAGCATGCATTAT | Frameshift -7 bp / Sp donor site -1 bp |  | No |
| **Del CTTCA** | ATTTACTGGGTCAGCCTGCAGAC---------TGGTAAGTTCAGCATGCATTAT | Frameshift -5 bp / Sp donor site -1 bp | Stop | No |
| **Del AGACCTTCA** | ATTTACTGGGTCAGCCTGC-----------------TGGTAAGTTCAGCATGCATTAT | In frame -9 bp / Sp donor site -1 bp | RPS/--- | No |
| **Del CAGACCTTCATGGTAAGTT** | ATTTACTGGGT------------------------------------------------CAGCATGCATTAT | In frame -12 bp / Sp donor site -7 bp | RPSC/---- | No |
| **G-C** | ATTTACTGGGTCAGCCTGCAGACCTTCATGCTAAGTTCAGCATGCATTAT | Sp donor site |  | No |
