# Supplementary material for: Splice donor site sgRNAs enhance CRISPR/Cas9-mediated knockout efficiency
Source: PLoS One. 2019 May 9;14(5):e0216674. doi: 10.1371/journal.pone.0216674 (PMC6508695; doi:10.1371/journal.pone.0216674)
Supplement: S4 Table — NGS analysis of allelic variants induced in Baf/3 mouse cells. (DOCX) [file pone.0216674.s004.docx]

**S4 Table.-** *In vitro* genome editing of the mouse *Atm* locus using sgRNA against the exon coding sequence (IE) and the coding SDE sequence. NGS analysis of allelic variants induced in Baf/3 mouse cells.

| **IE-*mAtm* sgRNA** | **Sequence** | **Mutation** | **Result** | **Protein translation** |
| --- | --- | --- | --- | --- |
| **WT** | GGATCAAAATTTGGTCTATTACCTTTCGTGGTATAAGTTCTGGACAAACA |  |  |  |
| **Del T** | GGATCAAAATTTGGTCTATTACCTT--CGTGGTATAAGTTCTGGACAAACA | Frameshift -1 bp | Stop | No |
| **Del ACCTT** | GGATCAAAATTTGGTCTATT-------TCGTGGTATAAGTTCTGGACAAACA | Frameshift -5 bp | Stop | No |
| **Del ACCT** | GGATCAAAATTTGGTCTATT------TTCGTGGTATAAGTTCTGGACAAACA | Frameshift -4 bp | Stop | No |
| **Del C** | GGATCAAAATTTGGTCTATTA-CTTTCGTGGTATAAGTTCTGGACAAACA | Frameshift -1 bp | Stop | No |
| **Ins CTT** | GGATCAAAATTTGGTCTATTACCTT**CTT**TCGTGGTATAAGTTCTGGACAAACA | In frame +3 bp | -/F | Yes |
| **Del CT** | GGATCAAAATTTGGTCTATTAC--TTCGTGGTATAAGTTCTGGACAAACA | Frameshift -2 bp | Stop | No |
| **SDE-*mAtm*sgRNA** | **Sequence (Splice site; Exon; Intron)** | **Mutation** | **Result** | **Protein translation** |
| **WT** | TACTGGCTCAGCCTGTAAACCTTCTAGGTAGGTTGTATAATATTTGATTT |  |  |  |
| **Ins A** | TACTGGCTCAGCCTGTAAACCTTCTA**A**GGTAGGTTGTATAATATTTGATTT | Frameshift +1 bp / Sp donor site |  | No |
| **Del AGGT** | TACTGGCTCAGCCTGTAAACCTTCTAGGT--------TGTATAATATTTGATTT | In frame / Sp donor site -2 bp |  | No |
| **Del G** | TACTGGCTCAGCCTGTAAACCTTCTAG--TAGGTTGTATAATATTTGATTT | Sp donor site -1 bp |  | No |
| **Del AG** | TACTGGCTCAGCCTGTAAACCTTCT----GTAGGTTGTATAATATTTGATTT | Frameshift -2 bp / Sp donor site -2 bp | Stop | No |
| **Del CT** | TACTGGCTCAGCCTGTAAACCTT----AGGTAGGTTGTATAATATTTGATTT | Frameshift -2 bp / Sp donor site -1 bp | Stop | No |
| **Del GGT** | TACTGGCTCAGCCTGTAAACCTTCTA------AGGTTGTATAATATTTGATTT | Frameshift -1 bp / Sp donor site -3 bp | Stop | No |
| **Del C** | TACTGGCTCAGCCTGTAAACCTT--TAGGTAGGTTGTATAATATTTGATTT | Frameshift -1 bp | Stop | No |
| **Del GTAGG** | TACTGGCTCAGCCTGTAAACCTTCTAG----------TTGTATAATATTTGATTT | In frame / Sp donor site -4 bp |  | No |
| **Del GGTAGGTTGTA** | TACTGGCTCAGCCTGTAAACCTTCTA---------------------TAATATTTGATTT | Frameshift -1 bp / Sp donor site -6 bp | Stop | No |
| **Del GGTAGG** | TACTGGCTCAGCCTGTAAACCTTCTA----------GTTGTATAATATTTGATTT | Frameshift -1 bp / Sp donor site -6 bp | Stop | No |
| **Del ggtaggttgtata** | TACTGGCTCAGCCTGTAAACCTTCTA-------------------------ATATTTGATTT | Frameshift -1 bp / Sp donor site -7 bp | Stop | No |
| **Del GTAGGTTG** | TACTGGCTCAGCCTGTAAACCTTCTAG----------------TATAATATTTGATTT | Sp donor site -4 bp |  | No |
