# Supplementary material for: Splice donor site sgRNAs enhance CRISPR/Cas9-mediated knockout efficiency
Source: PLoS One. 2019 May 9;14(5):e0216674. doi: 10.1371/journal.pone.0216674 (PMC6508695; doi:10.1371/journal.pone.0216674)
Supplement: S5 Table — (DOCX) [file pone.0216674.s005.docx]

**S5 Table.-** NGS analysis of *ATM* allelic variants induced in human K562 single-edited cell-derived clones.

| **CLONE** | **IE-*hATM*sgRNA** | **Sequence** | **Mutation** | **Result** | **Protein translation** |
| --- | --- | --- | --- | --- | --- |
|  | WT | ACAGCGACATGGGGAACGTACACCATATGTGTTACGATGCCTTACGGAAG |  |  |  |
| **7** | Del TATG | ACAGCGACATGGGGAACGTACACCA-------TGTTACGATGCCTTACGGAAG | Frameshift -4 bp | Stop | No |
|  | Del GTGTTACGATGCCTTACGG | ACAGCGACATGGGGAAC------------------------------------------------------AAG | Frameshift -19 bp | Stop | No |
| **8** | del GTGTTAC | ACAGCGACATGGGGAACGTACACCATAT-------------GATGCCTTACGGAAG | Frameshift -7 bp | Stop | No |
|  | Del ACCATATGTGTT | ACAGCGACATGGGGAACGTAC----------------------ACGATGCCTTACGGAAG | In frame -12 bp | PYVL/---- | Yes |
| **9** | WT | ACAGCGACATGGGGAACGTACACCATATGTGTTACGATGCCTTACGGAAG |  |  | Yes |
|  | Del TATGTG | ACAGCGACATGGGGAACGTACACCA-----------TTACGATGCCTTACGGAAG | In frame -6 bp | YV/-- | Yes |
| **10** | WT | ACAGCGACATGGGGAACGTACACCATATGTGTTACGATGCCTTACGGAAG |  |  | Yes |
|  | WT | ACAGCGACATGGGGAACGTACACCATATGTGTTACGATGCCTTACGGAAG |  |  | Yes |
| **11** | Del AT | ACAGCGACATGGGGAACGTACA---------TGTGTTACGATGCCTTACGGAAG | Frameshift -2 bp | Stop | No |
|  | Del TACACCATATGTGT | ACAGCGACATGGGGAACG-------------------------TACGATGCCTTACGGAAG | Frameshift -14 bp | Stop | No |
| **12** | WT | ACAGCGACATGGGGAACGTACACCATATGTGTTACGATGCCTTACGGAAG |  |  | Yes |
|  | Del ACCATATGTGTTACGATGCCTTACGGA | ACAGCGACATGGGGAACGTAC------------------------------------------------AG | In frame -27 bp | YVLRCLTE/-------- | Yes |
| **CLONE** | **SDE-*hATM*sgRNA** | **Sequence (Splice site; Exon; Intron)** | **Mutation** | **Result** | **Protein translation** |
|  | WT | ATTTACTGGGTCAGCCTGCAGACCTTCATGGTAAGTTCAGCATGCATTAT |  |  |  |
| **13** | Ins T | ATTTACTGGGTCAGCCTGCAGACCTTCA**T**TGGTAAGTTCAGCATGCATTAT | Frameshift +1 bp / SP donor site +1 bp | Stop | No |
|  | Del ATGGT | ATTTACTGGGTCAGCCTGCAGACCTTC----------AAGTTCAGCATGCATTAT | In frame -3 bp / SP donor site -5 bp | W/- | No |
| **14** | Del TGCAGACCTTCA | ATTTACTGGGTCAGCC----------------------TGGTAAGTTCAGCATGCATTAT | In frame -12 bp / SP donor site -1 bp | CRPS/---- | No |
|  | Del ATG | ATTTACTGGGTCAGCCTGCAGACCTTC------GTAAGTTCAGCATGCATTAT | In frame -3 bp / SP donor site -3 bp | W/- | No |
| **15** | Ins T | ATTTACTGGGTCAGCCTGCAGACCTTCAT**T**GGTAAGTTCAGCATGCATTAT | Frameshift +1 bp / SP donor site +1 bp |  | No |
|  | Ins T | ATTTACTGGGTCAGCCTGCAGACCTTCAT**T**GGTAAGTTCAGCATGCATTAT | Frameshift +1 bp / SP donor site +1 bp |  | No |
| **16** | Del GTCAGCCTGCAGACCTTCATGGTAAGT | ATTTACTGG--------------------------------------------------TCAGCATGCATTAT | In frame -21 bp / SP donor site -7 bp |  | No |
|  | Del CTTCA | ATTTACTGGGTCAGCCTGCAGAC---------TGGTAAGTTCAGCATGCATTAT | Frameshift -5 bp / SP donor site -1 bp |  | No |
| **17** | Del CA | ATTTACTGGGTCAGCCTGCAGACCTT----TGGTAAGTTCAGCATGCATTAT | Frameshift -2 bp / SP donor site -1 bp |  | No |
|  | Del TGCAGACCTTCA | ATTTACTGGGTCAGCC----------------------TGGTAAGTTCAGCATGCATTAT | In frame – 12 bp / SP donor site -1 bp | CRPS/----- | No |
| **18** | WT | ATTTACTGGGTCAGCCTGCAGACCTTCATGGTAAGTTCAGCATGCATTAT |  |  | Yes |
|  | Del TGCAGACCTTCATGGTAAGTTCAGCA | ATTTACTGGGTCAGCC-----------------------------------------------TGCATTAT | Frameshift -14 bp / SP donor site -7 bp |  | No |
