# Supplementary material for: Splice donor site sgRNAs enhance CRISPR/Cas9-mediated knockout efficiency
Source: PLoS One. 2019 May 9;14(5):e0216674. doi: 10.1371/journal.pone.0216674 (PMC6508695; doi:10.1371/journal.pone.0216674)
Supplement: S6 Table — NGS analysis of allelic variants induced in microinjected mouse blastocysts. (DOCX) [file pone.0216674.s006.docx]

**S6 Table.-** *In vivo* genome editing of *Tyr* locus in mouse embryos using sgRNA against the coding sequence (IE) and the SDE sequence. NGS analysis of allelic variants induced in microinjected mouse blastocysts.

| **IE-*mTyr*sgRNA** | **Sequence** | **Mutation** | **Result** | **Protein translation** |
| --- | --- | --- | --- | --- |
| Del (TGCTCAG) | TTTATAATAGGACCTGCCAG-------------GCAACTTCATGGGTTTCAACTGC | Frameshift -7 bp | Stop | No |
| Del (CAGTGC) | TTTATAATAGGACCTGC-----------TCAGGCAACTTCATGGGTTTCAACTGC | In frame -6 bp | QC/-- | Yes |
| Del (AGTGC) | TTTATAATAGGACCTGCC----------TCAGGCAACTTCATGGGTTTCAACTGC | Frameshift -5 bp | Stop | No |
| Del (GCTCAGGCAACTTCATGGGTTTCAACTGCGGAAAGTGT) | TTTATAATAGGACCTGCCAGT---------------------------------------------------- | Frameshift -38 bp | Stop | No |
| Ins A | TTTATAATAGGACCTGCCAGTG**A**CTCAGGCAACTTCATGGGTTTCAACTGC | Frameshift +1 bp | Stop | No |
| Del (GCCAGTGCT) | TTTATAATAGGACCT-----------------CAGGCAACTTCATGGGTTTCAACTGC | In frame -9 bp | CQC/-- | Yes |
| Del (TCAGGC) | TTTATAATAGGACCTGCCAGTGC-----------AACTTCATGGGTTTCAACTGC | In frame -6 bp | SG/-- | Yes |
| **SDE-*mTyr*sgRNA** | **Sequence (Splice site; Exon; Intron)** | **Mutation** | **Result** | **Protein translation** |
| **WT** | AGCCCAGCATCCTTCTTCTCCTCCTGGCAGGTAAGATGCACTATATAGAG |  |  |  |
| Del CAGG | AGCCCAGCATCCTTCTTCTCCTCCTGG--------TAAGATGCACTATATAGAG | In frame -3 bp/ Sp donor site -4 bp | Q/-- | No |
| Del GGTAAGATGCA | AGCCCAGCATCCTTCTTCTCCTCCTGGCA---------------------CTATATAGAG | Frameshift -1bp / Sp donor site -4 bp | Stop | No |
| Del GCAGG | AGCCCAGCATCCTTCTTCTCCTCCTG----------TAAGATGCACTATATAGAG | Frameshift -4 bp / Sp donor site -4bp | Stop | No |
| Ins GG | AGCCCAGCATCCTTCTTCTCCTCCTGGC**GG**AGGTAAGATGCACTATATAGAG | Frameshift +2bp / Sp donor site +2 bp | Stop | No |
| Del CA | AGCCCAGCATCCTTCTTCTCCTCCTGG----GGTAAGATGCACTATATAGAG | Frameshift -2 bp / Sp donor site -2bp | Stop | No |
| Del CTGGCAGG | AGCCCAGCATCCTTCTTCTCCTC---------------TAAGATGCACTATATAGAG | Frameshift - 7 bp / Sp donor site -4bp | Stop | No |
| Del CAG | AGCCCAGCATCCTTCTTCTCCTCCTGG------GTAAGATGCACTATATAGAG | In frame -3 bp/ SP donor site -3 bp | Q/-- | No |
| G-T + Del TAA | AGCCCAGCATCCTTCTTCTCCTCCTGGCA**T**G------GATGCACTATATAGAG | In frame / Sp donor site -3bp |  | No |
