# Supplementary material for: Splice donor site sgRNAs enhance CRISPR/Cas9-mediated knockout efficiency
Source: PLoS One. 2019 May 9;14(5):e0216674. doi: 10.1371/journal.pone.0216674 (PMC6508695; doi:10.1371/journal.pone.0216674)
Supplement: S7 Table — Observed phenotype and Sanger analysis of allelic variants induced in mice born after CRISPR/Cas9 system microinjection. (DOCX) [file pone.0216674.s007.docx]

**S7 Table**.- *In vivo* genome editing of *Tyr* locus in mice using sgRNA against the coding sequence (IE) and the coding SDE sequence. Observed phenotype and Sanger analysis of allelic variants induced in mice born after CRISPR/Cas9 system microinjection.

| **IE-*mTyr*sgRNA** | | **Sequence** | **Mutation** | **Result** | **Protein translation** |
| --- | --- | --- | --- | --- | --- |
| WT | | TTTATAATAGGACCTGCCAGTGCTCAGGCAACTTCATGGGTTTCAACTGC |  |  |  |
| **Black** | WT | TTTATAATAGGACCTGCCAGTGCTCAGGCAACTTCATGGGTTTCAACTGC |  |  | Yes |
| WT | | TTTATAATAGGACCTGCCAGTGCTCAGGCAACTTCATGGGTTTCAACTGC |  |  | Yes |
| **Black** | WT | TTTATAATAGGACCTGCCAGTGCTCAGGCAACTTCATGGGTTTCAACTGC |  |  | Yes |
| Del GTGCTCAGGCA | | TTTATAATAGGACCTGCCA--------------------ACTTCATGGGTTTCAACTGC | Frameshift -11bp | Stop | No |
| **Black** | WT | TTTATAATAGGACCTGCCAGTGCTCAGGCAACTTCATGGGTTTCAACTGC |  |  | Yes |
| Del TGCTCAG | | TTTATAATAGGACCTGCCAG-------------GCAACTTCATGGGTTTCAACTGC | Frameshift -7 bp | Stop | No |
| **Black** | WT | TTTATAATAGGACCTGCCAGTGCTCAGGCAACTTCATGGGTTTCAACTGC |  |  | Yes |
|  | Del CAGTGC | TTTATAATAGGACCTGC-----------TCAGGCAACTTCATGGGTTTCAACTGC | In frame -6 bp | QC/-- | Yes |
| **Black** | WT | TTTATAATAGGACCTGCCAGTGCTCAGGCAACTTCATGGGTTTCAACTGC |  |  | Yes |
|  | Del TGCTCAG | TTTATAATAGGACCTGCCAG------------GCAACTTCATGGGTTTCAACTGC | Frameshift -7 bp |  | No |
|  | Ins A | TTTATAATAGGACCTGCCAGT**A**GCTCAGGCAACTTCATGGGTTTCAACTGC | Frameshift +1 bp |  | No |
| **Black** | WT | TTTATAATAGGACCTGCCAGTGCTCAGGCAACTTCATGGGTTTCAACTGC |  |  | Yes |
|  | WT | TTTATAATAGGACCTGCCAGTGCTCAGGCAACTTCATGGGTTTCAACTGC |  |  | Yes |
| **SDE-*mTYR*sgRNA** | | **Sequence (Splice site; Exon; Intron)** | **Mutation** | **Result** | **Protein translation** |
| WT | | AGCCCAGCATCCTTCTTCTCCTCCTGGCAGGTAAGATGCACTATATAGAG |  |  |  |
| **Mosaic** | WT | AGCCCAGCATCCTTCTTCTCCTCCTGGCAGGTAAGATGCACTATATAGAG |  |  | Yes |
|  | Del CAGGTA | AGCCCAGCATCCTTCTTCTCCTCCTGG------------AGATGCACTATATAGAG | In frame -3 bp / Sp donor site -6 bp | Q/- | No |
|  | Del G | AGCCCAGCATCCTTCTTCTCCTCCTGGCAG--TAAGATGCACTATATAGAG | Sp donor site -2bp |  | No |
| **Black** | WT | AGCCCAGCATCCTTCTTCTCCTCCTGGCAGGTAAGATGCACTATATAGAG |  |  | Yes |
|  | WT | AGCCCAGCATCCTTCTTCTCCTCCTGGCAGGTAAGATGCACTATATAGAG |  |  | Yes |
| **Mosaic** | WT | AGCCCAGCATCCTTCTTCTCCTCCTGGCAGGTAAGATGCACTATATAGAG |  |  | Yes |
|  | Del GGTAA | AGCCCAGCATCCTTCTTCTCCTCCTGGCA----------GATGCACTATATAGAG | Frameshift -1 bp / Sp donor site -5 bp |  | No |
|  | Ins A | AGCCCAGCATCCTTCTTCTCCTCCTGGCA**A**GGTAAGATGCACTATATAGAG | Frameshift +1 bp / Sp donor site +1 bp |  | No |
| **Mosaic** | WT | AGCCCAGCATCCTTCTTCTCCTCCTGGCAGGTAAGATGCACTATATAGAG |  |  | Yes |
|  | Del CAGGTAAG | AGCCCAGCATCCTTCTTCTCCTCCTGG----------------ATGCACTATATAGAG | In frame -3 bp / Sp donor site -7 bp | Q/- | No |
|  | Del GG / Ins CAT | AGCCCAGCATCCTTCTTCTCCTCCTGGC**CAT**GTAAGATGCACTATATAGAG | Sp donor site |  | No |
| **Albino** | Del A | AGCCCAGCATCCTTCTTCTCCTCCTGGC--GGTAAGATGCACTATATAGAG | Frameshift -1 bp / Sp donor site -1bp |  | No |
|  | Del CCTGGCAGGTA / Ins ATAT | AGCCCAGCATCCTTCTTCTCCT**ATATA**-----------AGATGCACTATATAGAG | In frame / Sp donor site |  | No |
