# Supplementary material for: Splice donor site sgRNAs enhance CRISPR/Cas9-mediated knockout efficiency
Source: PLoS One. 2019 May 9;14(5):e0216674. doi: 10.1371/journal.pone.0216674 (PMC6508695; doi:10.1371/journal.pone.0216674)
Supplement: S8 Table — NGS analysis of allelic variants induced in K562 cells. (DOCX) [file pone.0216674.s008.docx]

**S8 Table.-** *In vitro* genome editing of the human ABL-1 locus using sgRNA against the exon coding sequence (IE) and the coding SDE sequence. NGS analysis of allelic variants induced in K562 cells.

| **IE-hABL-1(TK3) sgRNA** | **Sequence** | **Mutation** | **Result** | **Protein translation** |
| --- | --- | --- | --- | --- |
| **WT** | ACTCAGATCTCGTCAGCCATGGAGTACCTGGAGAAGAAAAACTTCATCCA |  |  |  |
| **Ins T** | ACTCAGATCTCGTCAGCCATGGAG**T**TACCTGGAGAAGAAAAACTTCATCCA | Frameshift +1 bp | Stop | No |
| **Del GT** | ACTCAGATCTCGTCAGCCATGGA----ACCTGGAGAAGAAAAACTTCATCCA | Frameshift -2 bp | Stop | No |
| **Del AC** | ACTCAGATCTCGTCAGCCATGGAGT----CTGGAGAAGAAAAACTTCATCCA | Frameshift -2 bp | Stop | No |
| **Del AGT** | ACTCAGATCTCGTCAGCCATGG------ACCTGGAGAAGAAAAACTTCATCCA | In frame -3 bp | EY/D | Yes |
| **Del GAGT** | ACTCAGATCTCGTCAGCCATG--------ACCTGGAGAAGAAAAACTTCATCCA | Frameshift -4 bp | Stop | No |
| **Del ATGGAGT** | ACTCAGATCTCGTCAGCC--------------ACCTGGAGAAGAAAAACTTCATCCA | Frameshift -7 bp | Stop | No |
| **Del TGGAGTACC** | ACTCAGATCTCGTCAGCCA-----------------TGGAGAAGAAAAACTTCATCCA | In frame -9 bp | EYL/-- | Yes |
| **Del GGA** | ACTCAGATCTCGTCAGCCAT------GTACCTGGAGAAGAAAAACTTCATCCA | In frame -3 bp | E/-- | Yes |
| **A>C** | ACTCAGATCTCGTCAGCCATGGAGT**C**CCTGGAGAAGAAAAACTTCATCCA | In frame | Y/S | Yes |
| **Del CCATGGAGTA** | ACTCAGATCTCGTCAG------------------CCTGGAGAAGAAAAACTTCATCCA | Frameshift -10 bp | Stop | No |
| **SDE-*hABL-1* sgRNA** | **Sequence (Splice site; Exon; Intron)** | **Mutation** | **Result** | **Protein translation** |
| **WT** | CTGACGGTGGCCGTGAAGACCTTGAAGGTAGGCTGGGACTGCCGGGGGTG |  |  |  |
| **Ins A** | CTGACGGTGGCCGTGAAGACCTTG**A**AAGGTAGGCTGGGACTGCCGGGGGTG | Frameshift +1 bp | Stop | No |
| **Del GAA** | CTGACGGTGGCCGTGAAGACCTT------GGTAGGCTGGGACTGCCGGGGGTG | In frame -3 bp / Sp donor Site -2 bp | K/-- | No |
| **Del G** | CTGACGGTGGCCGTGAAGACCTTGAA--GTAGGCTGGGACTGCCGGGGGTG | Frameshift -1 bp / Sp donor Site -1 bp | Stop | No |
| **Del AA** | CTGACGGTGGCCGTGAAGACCTTG----GGTAGGCTGGGACTGCCGGGGGTG | Frameshift -2 bp / Sp donor Site -2 bp | Stop | No |
| **A>G** | CTGACGGTGGCCGTGAAGACCTTGA**G**GGTAGGCTGGGACTGCCGGGGGTG | In frame / Sp donor Site | K/R | No |
| **Del A** | CTGACGGTGGCCGTGAAGACCTTG--AGGTAGGCTGGGACTGCCGGGGGTG | Frameshift -1 bp / Sp donor Site -1 bp | Stop | No |
| **G>C** | CTGACGGTGGCCGTGAAGACCTTGAA**C**GTAGGCTGGGACTGCCGGGGGTG | In frame / Sp donor Site | K/N | No |
| **A>C** | CTGACGGTGGCCGTGAAGACCTTGA**C**GGTAGGCTGGGACTGCCGGGGGTG | In frame / Sp donor Site | K/T | No |
| **Del TGAA** | CTGACGGTGGCCGTGAAGACCT-------GGTAGGCTGGGACTGCCGGGGGTG | Frameshift -4 bp / Sp donor Site -2 bp | Stop | No |
