# Supplementary material for: Splice donor site sgRNAs enhance CRISPR/Cas9-mediated knockout efficiency
Source: PLoS One. 2019 May 9;14(5):e0216674. doi: 10.1371/journal.pone.0216674 (PMC6508695; doi:10.1371/journal.pone.0216674)
Supplement: S9 Table — (DOCX) [file pone.0216674.s009.docx]

**S9 Table**.- Oligos designed for each sgRNA.

|  | **Forward** | **Reverse** |
| --- | --- | --- |
| **IE-*mTyr*sgRNA** | CACCGAATAGGACCTGCCAGTGCTC | AAACGAGCACTGGCAGGTCCTATTC |
| **SDE-*mTyr*sgRNA** | CACCGTATAGTGCATCTTACCTGCC | AAACGGCAGGTAAGATGCACTATAC |
| **IE-*hTYR*sgRNA** | CACCGGTGTCAATGGATGCACTGCT | AAACAGCAGTGCATCCATTGACACC |
| **SDE-*hTYR*sgRNA** | CACCGATCATTCTTCTCCTCTTGGC | AAACGCCAAGAGGAGAAGAATGATC |
| **IE-*mAtm*sgRNA** | CACCGATTTGGTCTATTACCTTTCG | AAACCGAAAGGTAATAGACCAAATC |
| **SDE-*mAtm*sgRNA** | CACCG TATTATACAACCTACCTAGA | AAACTCTAGGTAGGTTGTATAATAC |
| **IE-*hATM*sgRNA** | CACCGCATTCCGTAGCATTGTGTAT | AAACATACACAATGCTACGGAATG |
| **SDE-*hATM*sgRNA** | CACCGGCATGCTGAACTTACCATGA | AAACTCATGGTAAGTTCAGCATGC |
| **IE-*hABL-1*sgRNA** | CACCCTCGTCAGCCATGGAGTACC | AAACGGTACTCCATGGCTGACGAG |
| **SDE-*hABL-1*sgRNA** | CACCGCCGTGAAGACCTTGAAGGT | AAACACCTTCAAGGTCTTCACGGCC |
