# Supplementary material for: Splice donor site sgRNAs enhance CRISPR/Cas9-mediated knockout efficiency
Source: PLoS One. 2019 May 9;14(5):e0216674. doi: 10.1371/journal.pone.0216674 (PMC6508695; doi:10.1371/journal.pone.0216674)
Supplement: S10 Table — (DOCX) [file pone.0216674.s010.docx]

**S10 Table**.- Oligos used for target genome sequence amplification.

|  | **Forward** | **Reverse** |
| --- | --- | --- |
| **IE-*mTyr*** | GTTCTTGGCTGTTTTGTATTGCC | AAACATGGGTGTTGACCCATTGT |
| **SDE-*mTyr*** | ATATGGAGGGACATTGATTTTG | GGAGGTGCTAACAATACAGTG |
| ***mAtm*** | GCCTTAAGGAAGTTGCCTTATG | CTACACAGGAAGATCCTAACTC |
| ***hATM*** | GGCTACAGATTGCAACCCAATTA | AAGTCTTCTCGGCCAAACAAGA |
| ***hTYR*** | TGAGTGCCCCAGAGAAGGAC | TGCAATGAGTGTTCAGGTGAGA |
| **IE-*hABL-1*** | AGTCAGAATCCTTCAGAAGGCT | CTGAATTTAGCCCTGGATGCAT |
| **SDE-*hABL-1*** | GCTGTTCCCTGTTTCCTTCAG | AATTCCGTGGCGCCAATGAAG |
