# Supplementary material for: Splice donor site sgRNAs enhance CRISPR/Cas9-mediated knockout efficiency
Source: PLoS One. 2019 May 9;14(5):e0216674. doi: 10.1371/journal.pone.0216674 (PMC6508695; doi:10.1371/journal.pone.0216674)
Supplement: S11 Table — (DOCX) [file pone.0216674.s011.docx]

**S11 Table** .- Oligos used for *in vitro* transcription of sgRNA and Cas9 mRNA.

|  |  |
| --- | --- |
| **T7 IE-m*Tyr*sgRNA F** | TAATACGACTCACTATAGGAATAGGACCTGCCAGTGCTC |
| **T7 SDE-m*Tyr*sgRNA F** | TAATACGACTCACTATAGGTATAGTGCATCTTACCTGCC |
| **gRNA R** | GCACCGACTCGGTGCCACT |
| **T7-Cas9 F** | TAATACGACTCACTATAGGGATGGCCCCAAAGAAGAAGCGGA |
| **Cas9 R** | CTTTTTCTTTTTTGCCTGGCC |
