# Supplementary material for: Splice donor site sgRNAs enhance CRISPR/Cas9-mediated knockout efficiency
Source: PLoS One. 2019 May 9;14(5):e0216674. doi: 10.1371/journal.pone.0216674 (PMC6508695; doi:10.1371/journal.pone.0216674)
Supplement: S12 Table — (DOCX) [file pone.0216674.s012.docx]

**S12 Table**.- Oligos used for off-target genome sequence amplification.

|  | **Target** | **Forward** | **Reverse** |  | **Target** | **Forward** | **Reverse** |
| --- | --- | --- | --- | --- | --- | --- | --- |
| **IE-m*Tyr* sgRNA** | *Crebbp* | GATGCATGTGTGGAGGTCAGA | AAGTGTGTATGTGTTCACATGTG | **SDE-m*Tyr* sgRNA** | *Urb5* | AGAACCTTAGGGTGGATGGAG | TGCAATTCAGTAAGTACCAATGTT |
|  | *Pcsk9* | GCAGTAGATTTGCAAAGCCCC | CTCTAAAGTCCCAAGCATGTGG |  | *Akap7* | TAGGCCACTTTTAATAAGTGTGC | ACAGAGGGCCTATGCACAGG |
|  | *Mgat5* | TAAGCACTGGCTGTCTGAGAG | GGAAATCACCCAGTGCTGACC |  | *Pygb* | AGGGTCACCATACCACATGAG | TGGGCACTACTACCAAAGCAC |
|  | *Olfr56* | ACTTCCTAACCACAGAGGCAC | CATTGTTAAGGACAGTCACTGTC |  | *Vmn2r109* | TCCTTACAAATACCCAGAAGACA | AGGTATAAAGCCACTCTCATTGT |
|  | *Setd7* | TTGATTCGCTTCCCTGAAGTCT | ATGGGGCTCTTGCCAGCATG |  | *Vmn2r110* | AACAGTTAATCCTTACAAATACCC | ATGTCTACTCATTTATTCCTGTTG |
| ***IE-hTYR* sgRNA** | MIR2052 | TCTTGAAGAGTTCAGCCAGTG | GTTGTTTGTCCCAAATCACATAG | ***SDE-*h*TYR* sgRNA** | *CABYR* | CAGAATGCAGCTGAACCTATTG | GGGGGGAGACCAACTTGGG |
|  | PTPN7 | GAGATCGCACCATGGCATTCC | TACCTGTGGGTGGGCTGGGA |  | *COL27A1* | GAGCCTCCACTCCACTCCC | ATGCAGCTCACCCTCCAGGA |
|  | NAV2 | ATGTTTTAAGTACTGAGATTGCTG | CCTTAATTCCCCTCGCCATGAA |  | *CCNJ* | AGCCATCATATTGTCTTTACTCCT | GAGATGGAGTCTTGCTCTGTC |
|  | CCSER1 | CCCTTGGCCCCTCCCAAATT | GGGGTTCGAGCCCCCACAT |  | *SMIM31* | TGCTCCCCAACCCACCCAC | GGAAGTCAGGAGAAAGAGTGG |
|  | PTPRZ1 | GTAAGTGAGAGCATGTGGC | AAGACATACAAATGGCAGATAGG |  | *WASF3* | CCACCTGCTGTTCTTTCACCC | GGCATCTGTGCTCATGGAGC |
| ***IE-*m*Atm* sgRNA** | *Proser2* | TCGGTGAGTTCCGCCCTACT | TGCCTAGAGGGGGTTAGTTAC | ***SDE-*m*Atm* sgRNA** | *Hdlbp* | GCCTGGGATAGGAATGTGATTG | AGAGGGATGATGATCTGAAGTG |
|  | *Gm22334* | TTTCTGCCACACATTTACAGGAA | TATGCGTTCTATATTCAAGTCCC |  | *Wdr44* | AGATTCCATCCTGTACTGTGG | GGTTCTATACCCCAGTATAAGG |
|  | *Fam208b* | CACTTAATGTTCTTGATCCTTGG | CCCTGTAAATACTTCTTTCTGGT |  | *D12rik* | TTGAAAGGGCTGGTGGCTGAT | AGATACAGCTCGATCAAGTTCC |
|  | *Npas3* | CATGGCAACTGGTTTGCAGAG | GACTTCATGGGATGGGGGAAG |  | *Usp13* | AGACTTACCCTATAATGGCTCC | AGCAGCCAGACATCAGTTCTC |
|  | *Wnt10a* | GACAGGACATAGACAGGATGG | CACACAGACATACATGCGAGC |  | *Pbx3* | GGCCAGGGTCTGTTTATCTTC | GGCCAGCCTGGTTTACAGAG |
| **IE-h*ATM* sgRNA** | *C1ORF87* | GCACAGCACAGAGGAGTAGAG | GCCTTCTTGAACAGGAGAAGTG | **SDE-h*ATM* sgRNA** | *AL031773.1* | AGGAGTGAACTGTCATCGTATC | GGAGCCTTCCCTCTTCCAAC |
|  | *RAPGEF2* | TATTGACCCTGAGTGCCAGTC | CTTCTAGACCTGTTCTTGACAG |  | *INHBA-AS1* | GTAGGGAGACAGGAATTGTAGG | ACCCCCTTATCTTAACTCAAACA |
|  | *NRAP* | TGGAGATAGGAGCAGGAGAGA | GGGGACAGACTCACCTCGC |  | *AP001347.1* | TCATTGCCAACAGAACTTTTCTG | CTCACATTTGCTGGCCATTGAC |
|  | *LEF1-AS1* | AGGCTGCAGTGCAATCCTCC | GTCATTTCTGTGTCTGTTTCTGT |  | *DET1* | ATGGACTTGGAAGGGGCAGG | CTCACTGCAACCTCTGCCTC |
|  | *GHR* | CAGGTGTGTGCTGAGTGACTG | TTGTCAACTCTTGACTCAGATAC |  | *RNU6-490P* | CCCAGCCATTGTTCCTAAAGC | ACCCGACTTTTATACATGGATAC |
| **IE-h*ABL-1* sgRNA** | *YWHAG* | CTCCATCTCGTCTCACTGTAAC | CCCGGCCTCAAATTTCTTTTGG | **SDE-h*ABL-1*sgRNA** | ANKH | GGGGGGATGAAAGAAACAACTG | GCCTCCCAAGTAGCTGGGAT |
|  | *SHANK2* | GAGACATCTTGCCTGTTGCTG | CCAGGTTTTTCAGAGCCCTAG |  | DAG1 | CGTCAGTCTGCAGGGTCAGG | CCAAACCCAAGGGAGACAAGG |
|  | *AGBL4* | CATAACAGAACTCCGCAAAACC | CTAGACACAGAGTCTTGCTCTG |  | OR2A3P | CAGATGGCAATGGAGATAAAGG | TAACTTTACTAGGAAACGTTCACAG |
|  | *PSTPIP1* | GTCTCCCACATGGCACAGATG | AGACACAGGCCTGCTGCACA |  | FRMD4A | GGCCTTTCTACTCAGGTCGG | TGGCTTAGAGAAAGGTCTTCTC |
|  | *MICALL1* | CAGCTCCTTTCTGACTCTGAC | ATACTGGGACACATAGGTCATG |  | PTPRN2 | GCAATTAGAGGGAAGGACGTG | AAGGGCTGAGGCCACACTGA |
